# Antibacterial Property and Cytotoxicity of a Poly(lactic acid)/Nanosilver-Doped Multiwall Carbon Nanotube Nanocomposite

**DOI:** 10.3390/polym9030100

**Published:** 2017-03-10

**Authors:** Chi-Hui Tsou, Wei-Hua Yao, Yi-Cheng Lu, Chih-Yuan Tsou, Chin-San Wu, Jian Chen, Ruo Yao Wang, Chaochin Su, Wei-Song Hung, Manuel De Guzman, Maw-Cherng Suen

**Affiliations:** 1 Material Corrosion and Protection Key Laboratory of Sichuan Province, College of Materials Science and Engineering, Sichuan University of Science and Engineering, Zigong 643000, China; jchenzg@aliyun.com; 2 Faculties of Biological and Chemical Engineering, Faculties of Materials Engineering, Science and Technology Innovation Center, Panzhihua University, Panzhihua 617000, China; 3 Department of Materials Science, Chulalongkorn University, Bangkok 10330, Thailand; 4 Department of Materials and Textiles, Oriental Institute of Technology, New Taipei City 22061, Taiwan; angelayao@mail.oit.edu.tw; 5 Department of Materials Science and Engineering, National Taiwan University of Science and Technology, Taipei 10607, Taiwan; b10104003@mail.ntust.edu.tw (Y.-C.L.); keymykimo@gmail.com (C.-Y.T.); 6 Faculty of Electronic and Electrical Engineering, Huaiyin Institute of Technology, Huan’an 223003, China; 7 Department of Applied Cosmetology, Kao Yuan University, Kaohsiung 82101, Taiwan; cws1222@cc.kyu.edu.tw; 8 Department of Molecular Science & Engineering, National Taipei University of Technology, Taipei 10608, Taiwan; steven5202001@yahoo.com.tw (R.Y.W.); f10913@ntut.edu.tw (C.-c.-S.); 9 R&D Center for Membrane Technology, Chung Yuan University, Taoyuan 32023, Taiwan; ounnam@gmail.com; 10 Department of Fashion Business Administration, Lee-Ming Institute of Technology, Taishan, New Taipei City 24305, Taiwan

**Keywords:** poly(lactic acid), nanosilver-doped multiwall carbon nanotube, antibacterial property, cytotoxicity, bionanocomposites

## Abstract

A novel method was used to synthesize a nanosilver-doped multiwall carbon nanotube (MWCNT-Ag), and subsequently, the novel poly(lactic acid) (PLA)- and MWCNT-Ag-based biocompatible and antimicrobial nanocomposites were prepared by melt blending. Based on energy dispersive X-ray spectrometry images, an MWCNT-Ag was successfully synthesized. The effect of the MWCNT-Ag on the PLA bionanocomposites was investigated by evaluating their thermal and mechanical properties, antifungal activity, and cytotoxicity. The nanocomposites exhibited a high degree of biocompatibility with the MWCNT-Ag content, which was less than 0.3 phr. Furthermore, tensile strength testing, thermogravimetric analysis, differential scanning calorimetry, and antibacterial evaluation revealed that the tensile strength, thermostability, glass transition temperature, and antibacterial properties were enhanced by increasing the MWCNT-Ag content. Finally, hydrolysis analysis indicated that the low MWCNT-Ag content could increase the packing density of PLA.

## 1. Introduction

The applications of biodegradable polymers have been extensively reported [1,2,3,4,5,6]. Poly(lactic acid) (PLA) is one of the most popular biopolymers because of its high biocompatibility, and it has been widely used in drug delivery, scaffolds for tissue engineering, and bone fixation [7]. However, PLA has some drawbacks, such as low thermal resistance and low flexibility [3,4,5,8] and is easily colonized by fungi and bacteria [9]. Therefore, strategies to enhance its mechanical and antibacterial properties [10,11] are required for the commercial utilization of these polymers and for broadening their scope of application while maintaining their biodegradable and ecofriendly properties.

Overall, PLA has lower mechanical properties, including lower thermal resistance, a lower crystallization rate, and lower flexibility, than other polymers, such as polyethylene, polypropylene, and polyethylene terephthalate. Therefore, strategies to improve its mechanical and thermal properties are necessary for commercial applicability. The most efficient strategy for improving its properties is reinforcement with nanomaterials [12,13,14,15,16,17], and various nanomaterials, such as sepiolite [13], carbon nanotubes [12], montmorillonite [13,14], zirconium phosphonate [13], graphene [15], and graphene oxide [16] have been used. PLA-based nanocomposites with varying content of multiwall carbon nanotubes (MWCNTs) have been suggested to for facilitating promising medical applications [17,18,19]. For example, Wang et al. [12] grafted poly(acrylic acid) onto MWCNTs to prepare modified MWCNTs to improve the expansion ratio of PLA after foaming with a chain extender. Park et al. [20] reported the isothermal crystallization behavior and mechanical properties of PLA/MWCNT nanocomposites; specifically, they found that the MWCNTs effectively improved the isothermal crystallization rate of the PLA through heterogeneous nucleation. Lalwani et al. [21] reinforced PLA with an MWCNT for bone tissue engineering, which considerably improved the mechanical properties compared with those of a neat PLA. Additionally, Lahiri et al. [22] revealed that, compared with a neat copolymer, a PLA/polycaprolactone copolymer loaded with an MWCNT could increase the viability of human osteoblasts because of the surface roughness that was produced during the loading. 

PLA not only requires improvement of its mechanical properties, but also requires improvement of its antimicrobial properties to prevent potential bacterial infections. However, MWCNTs do not exhibit antimicrobial properties. Therefore, we synthesized a novel MWCNT-Ag to simultaneously provide antibacterial properties and improve the mechanical properties of PLA. The content of the MWCNT-Ag also decreased the biodegradable property of PLA, to enhance its stability for use in bone engineering. This study is the first to report a PLA/MWCNT-Ag nanocomposite, which might have potential biomedical applications. Our results also indicate the optimal content of MWCT-Ag in nanocomposites.

## 2. Experimental

### 2.1. MWCNT-Ag Synthesis

**Figure 1:**
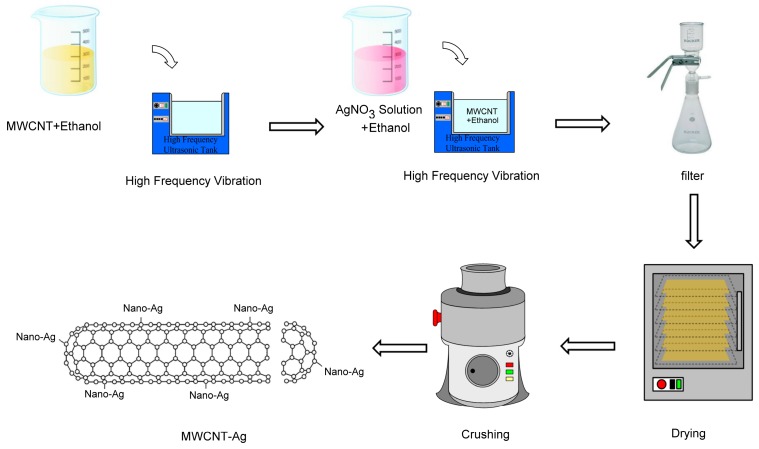
Nanosilver-doped multiwall carbon nanotube (MWCNT-Ag) synthesis.

The MWCNT used in this study was supplied by Apex Nanotek Corporation (New Taipei City, Taiwan), was N95% (see Figure S1a), and had a length and outer diameter of 6.5 ± 5.5 μm and 15 ± 5 nm, respectively. Silver nitrate (AgNO_3_; reagent grade, purity ≥99%, Sigma Aldrich, Saint Louis, MO, USA) was used as the Ag salt. The Ag nanoparticles were capped on the surface of the MWCNT by using the wetting dispersion method [23]. The Ag to MWCNT ratio was 1:9. First, 6.3 g of pure AgNO_3_ was dissolved in 1000 mL of ethanol (purity, 98%), and 39.6 g of MWCNT was dissolved in another 1000 mL of ethanol. Second, the MWCNT–ethanol solution was placed in an ultrasonic bath (Bransonic Ultrasonic Cleaner, 3510r-DTH, Branson, Danbury, CT, USA) for 30 min. Subsequently, the AgNO_3_-ethanol solution was poured into the MWCNT-ethanol solution, and the combined solution was placed in an ultrasonic bath for 120 min to avoid aggregation. The MWCNT and AgNO_3_ both dispersed and integrated well. This solution was passed through a filter to remove the ethanol; subsequently, the mixture was placed in an oven at 120 °C for 2 h to evaporate the residual ethanol. Finally, the mixture was crushed in a crushing machine (Chuanghua Corp., Dongguan, China) to obtain MWCNT-Ag (see Figure S1b). Figure 1 shows the synthesis of MWCNT-Ag. 

### 2.2. Preparation of PLA/MWCNT-Ag Nanocomposites

In the present study, PLA (Biopolymer 4032D) was obtained from Cargill-Dow and vacuum-dried in an oven at 80 °C for 8 h to remove the residual water. The PLA (50 g) was then melt-blended with MWCNT-Ag (0.05–0.015 g) using a Brabender mixer, which was operated at 190 °C with a screw speed of 120 rpm for 4 min. Subsequently, all nanocomposites were hot-pressed at 190 °C and 12 MPa for 2 min and then cooled in air at 24 °C. The nanocomposites were dark black despite the very low MWCNT-Ag content, because a large area of the MWCNT surface was available for dispersion in the nanocomposites (see Figure S2). Table 1 presents the compositions of the prepared nanocomposites. Similarly to the original PLA, the nanocomposites were vacuum-dried in an oven at 80 °C for 8 h before hot pressing. 

### 2.3. Thermogravimetric Analysis

Thermogravimetric analysis (TGA) was performed using a PerkinElmer TGA (model Pyris 1). The nanocomposite samples (8–10 mg) were heated from 105 to 600 °C under nitrogen at a rate of 10 °C/min.

### 2.4. Differential Scanning Calorimetry

Differential scanning calorimetry (DSC) was performed using a DSC (model Jade, PerkinElmer, Buckinghamshire, UK). The nanocomposites samples were sealed in an aluminum pan. Scans (55–185 °C) were performed at a heating rate of 10 °C/min under nitrogen purging. The maximum peak in the second scan of the endothermic transition was recorded as the melting point; samples of 7–8 mg in size were used for all of the scans.

### 2.5. Mechanical Properties

The tensile properties of the hot-pressed PLA and PLA/MWCNT-Ag nanocomposites at 25 °C were determined using a Shimadzu tensile testing machine (model AG-10KNA, Shimadzu, Tokyo, Japan) with a crosshead speed of 50 mm/min. A 35-mm dog-bone-shaped gauge was used for the tensile experiment, and these specimens were prepared according to the ASTM D638 Type IV standard. The tensile strength and elongation at break were based on the average tensile results of at least five specimens.

### 2.6. Antibacterial Evaluation

The antibacterial activity of PLA and PLA/MWCNT-Ag nanocomposites was examined against *Staphylococcus aureus*, an aerobic bacterium commonly observed in burn wounds. In the quantitative test, each nanocomposite was inoculated with a cell suspension (0.1 mL) at 30 °C for 24 h. The film was then transferred to a sterile physiological saline solution, and shaken and washed for 15 min in an aqueous bath. The bacterial suspension was diluted with the sterile physiological saline solution and inoculated onto a sterile Petri plate, which was subsequently incubated at 30 °C for 24 h. Finally, the grown colonies were counted.

### 2.7. Energy Dispersive X-ray Spectrometery

An energy dispersive X-ray spectrometer (EDX; model 7021-H Horiba EDX, Horiba, Kyoto, Japan) was used to assess the distribution of Ag content in the MWCNT and MWCNT-Ag nanocomposites. Gold-plated specimens of 1–2 mm^2^ were placed in the EDX chamber, which operated at 10 kV.

### 2.8. In Vitro Cytotoxicity Test (ISO 10993-5)

The PLA- and PLA/MWCNT-Ag nanocomposite-loaded plates were sterilized using an alcohol blast burner. First, all of the specimens were sprayed with alcohol and placed under the hood for overnight UV exposure. For sample extraction, the plates were treated with Dulbecco’s Modified Eagle’s Medium (DMEM) (0.2 g/mL) for 24 h at 37 °C in a 5% CO_2_ incubator. The phenol solution (0.64% *v*/*v*, Kelong Chemical Reagent Factory, ChengDu, China) was used as a positive control, and DMEM was used as a negative control. Next, third-passage rat fibroblasts were suspended at a concentration of 1 × 10^4^ cells/mL, and the cell suspensions were inoculated in 96-well plates (200 μL/well). Cell culture medium and L929 fibroblasts were purchased from the Food Industry Research and Development Institute (Hsinchu, Taiwan). Similar to the process for sample extraction, the cells were cultured in a 5% CO_2_ incubator at 37 °C for 24 h. Subsequently, the culture medium was removed and replaced by sample extracts and negative and positive control solutions. Thereafter, the cells were again cultured for 24 h, and analyzed using the methyl tetrazolium (MTT; Sigma, Saint Louis, MO, USA) assay after 24, 48, and 72 h of incubation; the fluid was recovered and analyzed without buffering, diluting, or filtering. After selecting eight wells, 20 μL of MTT dye (5 mg/mL) was added to each well, after which they underwent another 4-h incubation 37 °C in 5% CO_2_. Subsequently, 200 μL of dimethyl sulphoxide solution (Sigma) was added to dissolve the formazan crystals. Images of the fibroblast cells were captured using a microscope (OLYMPUS-CK30, OLYMPUS, Hamburg, Germany). The optical density (OD) of the formazan solutions was measured at 570 nm on an enzyme-linked immunosorbent assay microplate reader (Sunrise, Tecan, Switzerland), and the proliferation ratio was defined as the increased proportion of cells compared with the control. Proliferation at 24, 48, and 72 h was calculated as follows:

Proliferation % = *A*_c_/*A*_o_ (1)

where *A*_c_ is the absorbance of specimens, and *A*_o_ is the absorbance of the control on day 0. 

Cell viability was quantitatively determined using the MTT assay [19]. The relative growth rate (RGR) of the cell culture was calculated as follows:

(2) $$\mathrm{RGR}=\frac{\mathrm{OD}\;\mathrm{Value}\;\mathrm{of}\;\mathrm{sample}}{\mathrm{OD}\;\mathrm{Value}\;\mathrm{of}\;\mathrm{Blank}\;}\times 100\%$$

### 2.9. Hydrolysis Test

The hydrolytic degradation of a specimen was examined in a phosphate-buffered saline solution at 37 °C. The specimens, with dimensions of 20 mm × 20 mm × 1 mm, were tested for several days, washed with distilled water, and then completely vacuum-dried in an oven at 105 °C for 1 h. The degree of degradation was determined by the weight loss, which was calculated using the formula:

(3) $$\mathrm{weight}\;\mathrm{loss}=\frac{W_{t}-W_{0}}{W_{0}}\times 100\%$$

where *W*_0_ is the dry weight before degradation, and *W*_t_ is the dry weight at time *t*.

### 2.10. Morphology Analysis

The morphology of the specimens after hydrolytic degradation was observed using a Hitachi scanning electron microscope (SEM; model SU1510, Hitachi, Tokyo, Japan). Specimens with dimensions of 20 mm × 20 mm × 1 mm were fixed on a sample holder using a conductive adhesive tape and subsequently coated with a thin layer of gold to improve image resolution. The samples were gold-coated at 15 kV for 15 s before SEM examinations and photographed at 1.00 K magnification and low voltage (2.1 kV). 

## 3. Results and Discussion

### 3.1. TGA

**Figure 2:**
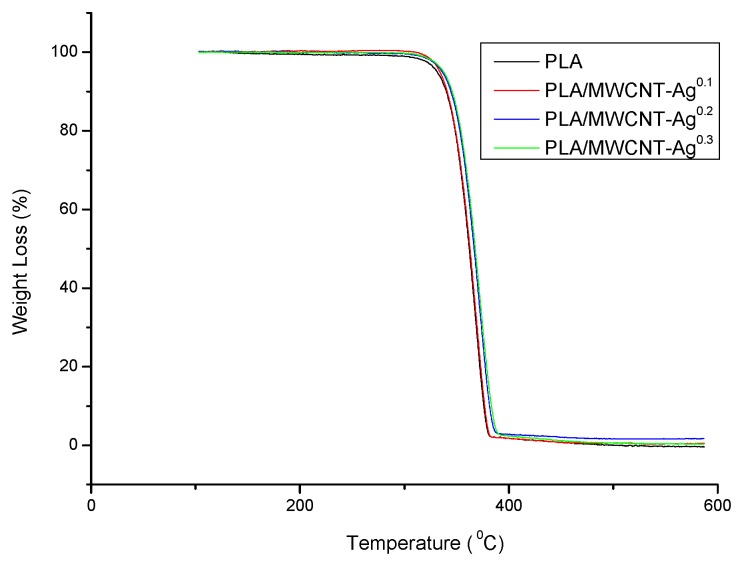
Thermogravimetric analysis (TGA) curves of the PLA and PLA/MWCNT-Ag nanocomposites (DTG curves are showed in Figure S3).

Figure 2 shows the TGA curves of the PLA and PLA/MWCNT-Ag nanocomposites, as well as the decomposition temperatures of all specimens that correspond to differential curves. Notably, the graph indicates that the addition of MWCNT-Ag improved the thermal stability of PLA. For instance, the temperature of the neat PLA was 330.9 °C for a weight loss of 5 wt %, and after 0.3 phr, the MWCNT-Ag was blended with the PLA and the temperature increased to 336.8 °C (a substantial increase of nearly 6 °C).

### 3.2. DSC

The glass transition temperature (*T*_g_), melting temperature (*T*_m_), and degree of crystallinity (*X*_c_) values from the DSC scans of the PLA and PLA/MWCNT-Ag nanocomposites are listed in Table 2. As noted earlier, Table 1 shows that *T*_g_ increased with more MWCNT-Ag content in the PLA/MWCNT-Ag nanocomposites, suggesting that *T*_g_ increased because MWCNT-Ag hindered the molecular chain motion in PLA. Furthermore, the *X*_c_ and *T*_m_ values increased significantly after the addition of MWCNT-Ag (see Table 1 and Figure S4), which indicates that MWCNT-Ag acts as a nucleating agent in the PLA/MWCNT-Ag nanocomposites. MWCNT-Ag induced heterogeneous nucleation, and low energy consumption was required to reach the critical stability value in the crystal range and growth [19]. 

### 3.3. Tensile Properties

**Figure 3:**
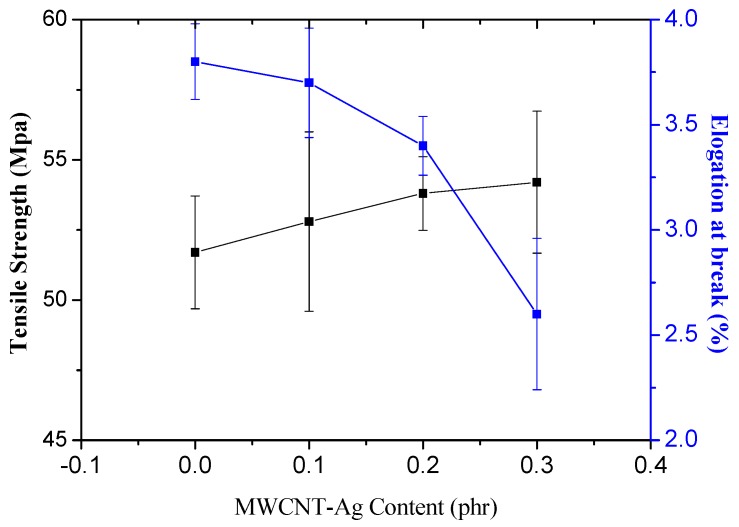
Tensile properties of the PLA and PLA/MWCNT-Ag nanocomposites.

Figure 3 shows the tensile strength and elongation at the break of the neat PLA and PLA/MWCNT-Ag nanocomposites. The tensile strength of the PLA was 51.7 Mpa; however, after the PLA was blended with MWCNT-Ag, the tensile strength of the PLA/MWCNT-Ag nanocomposites was substantially increased. For instance, the tensile strength of the PLA/MWCNT-Ag nanocomposites increased from 51.7 to 54.2 MPa when their MWCNT-Ag content increased from 0 to 0.3 phr. This improvement occurred because uniform dispersion increases the MWCNT-Ag surface area available for bonding with the polymer matrix. The enhancement of tensile strength for PLA/MWCNT-Ag nanocomposites can be adopted for bone tissue engineering or bone nails applications. However, the increasing rigidity of the PLA might be not easy to use in biomedical materials. Therefore, it is suggested that MWCNT-Ag content be equal to 0.2 phr, because the tensile strength is similar to that of MWCNT-Ag content at 0.3 phr but the elongation at the break is significantly reduced (0.3 phr). Adding a toughener in PLA/MWCNT-Ag nanocomposites is a worthwhile topic for future research.

### 3.4. Antibacterial Evaluation

**Figure 4:**
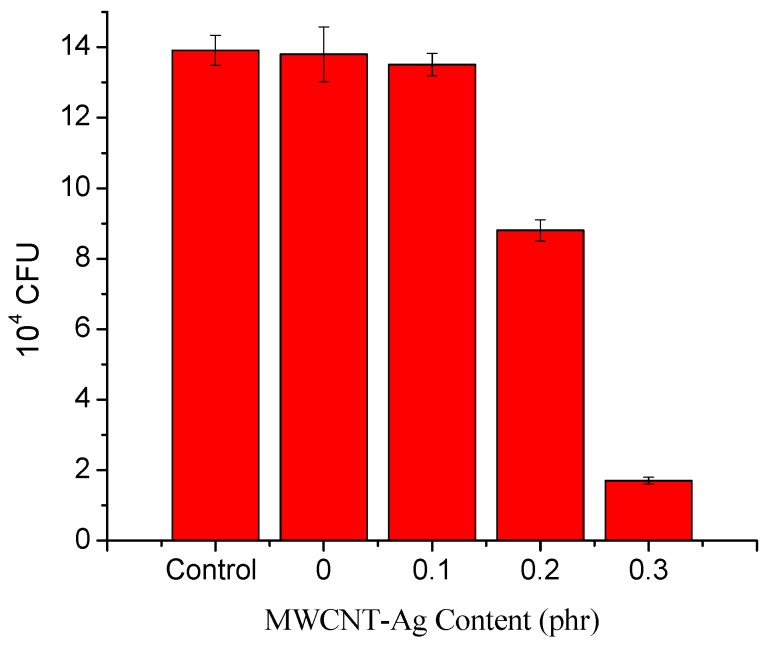
Antibacterial activity of the PLA and PLA/MWCNT-Ag nanocomposites.

Figure 4 shows the antibacterial activity of the neat PLA and PLA/MWCNT-Ag nanocomposites. Specifically, we found that the antibacterial activity decreased with increasing MWCNT-Ag content in PLA/MWCNT-Ag nanocomposites. Moreover, the MWCNT-Ag could import antimicrobial properties to the PLA; the nanoAg to MWCNT ratio was 1:9 and the nanoAg content in PLA was only 0.01–0.03 phr. Thus, despite the extremely low antibacterial agent content, the PLA acquired antimicrobial properties. 

### 3.5. Elemental Composition of MWCNT-Ag and Distribution of Ag in the PLA/MWCNT-Ag Nanocomposites

**Figure 5:**
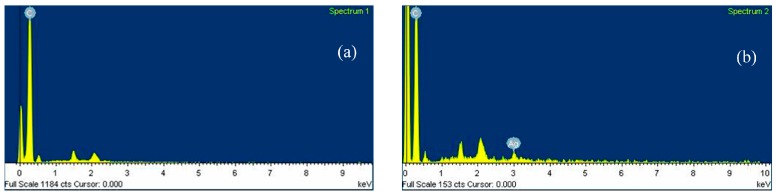
EDX image of (**a**) MWCNT and (**b**) MWCNT-Ag.

**Figure 6:**
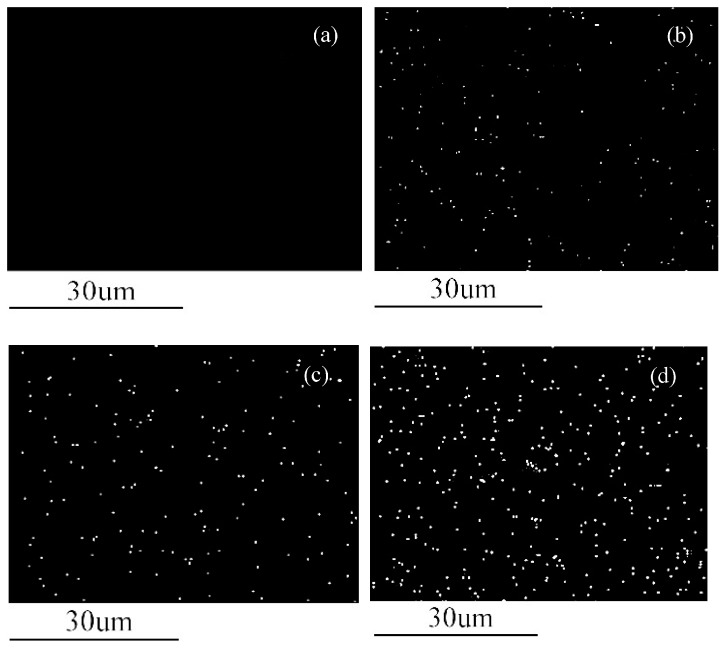
EDX images of Ag distribution in the PLA/MWCNT-Ag nanocomposites: (**a**) PLA; (**b**) PLA/MWCNT-Ag^0.1^; (**c**) PLA/MWCNT-Ag^0.2^; and (**d**) PLA/MWCNT-Ag^0.3^.

Table 3 provides the elemental composition details of MWCNT and MWCNT-Ag. In addition, Figure 5 shows the EDX images of the MWCNT and MWCNT-Ag reviewed in the present study, which indicate that the weight ratio of nanoAg to MWCNT approached 1:9. The EDX mapping in Figure 6 illustrates the distribution of elemental Ag as a function of the increasing MWCNT-Ag content; the white spots in the images represent nanoAg, which is uniformly distributed in the nanocomposites. As expected, no white spots were noted in the neat PLA, and the white spot distribution increased alongside increasing MWCNT-Ag content. These findings demonstrate that the antibacterial properties of PLA are attributed to the presence of nanoAg and its uniform distribution in the nanocomposites. As noted in the preceding section, PLA has been applied as bone nails or in bone tissue engineering; therefore, PLA not only requires tensile strength but must also possess antibacterial properties to combat bacterial infections and be successfully implanted in the human body.

### 3.6. Cytotoxicity Test

**Figure 7:**
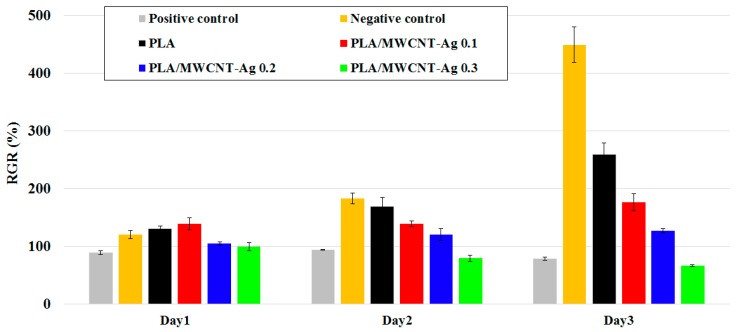
Methyl tetrazolium (MTT) assay results for the PLA and PLA/MWCNT-Ag nanocomposites.

**Figure 8:**
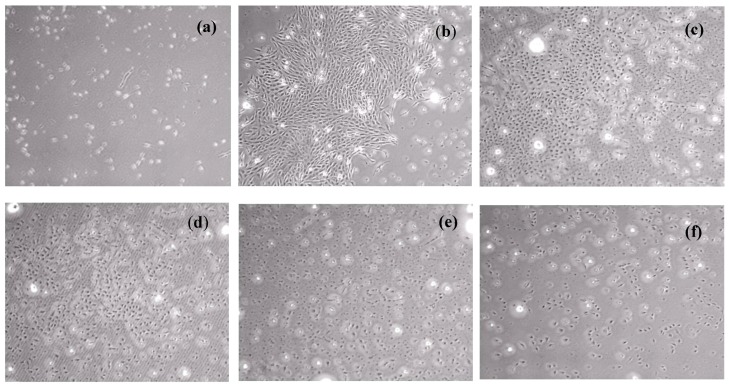
L929 fibroblasts cultured for three days with (**a**) positive control; (**b**) negative control; (**c**) PLA; (**d**) PLA/MWCNT-Ag^0.1^; (**e**) PLA/MWCNT-Ag^0.2^; and (**f**) PLA/MWCNT-Ag^0.3^. (L929 fibroblasts cultured for control is showed in Figure S5; for two days are showed in Figure S6).

The results of the MTT assay for neat PLA and PLA/MWCNT-Ag nanocomposites are shown in Figure 7 and Figure 8. Specifically, the MTT assay revealed that the RGRs of PLA, PLA/MWCNT-Ag^0.1^, and PLA/MWCNT-Ag^0.2^ after 72 h of incubation were 258.76%, 176.29%, and 126.39%, respectively. These specimens also had a cytotoxicity score of grade 0 (ISO 10993-5) [24,25], indicating no cytotoxicity. L929 fibroblasts exhibited a normal proliferation rate after incubation, with extracts of the PLA with low MWCNT-Ag content (0.1–0.2 phr). The RGR of the PLA/MWCNT-Ag nanocomposites with 0.3 phr of MWCNT-Ag was 65.98%, indicating very low cytotoxicity; however, the RGR decreased with an increase in the MWCNT-Ag content, which may be because of MWCNT-Ag release. Overall, the MTT assay revealed that the nanocomposites (MWCNT-Ag content: 0.1–0.2 phr) synthesized in the present study have high biocompatibility.

### 3.7. Hydrolysis of the PLA/MWCNT-Ag Nanocomposites

**Figure 9:**
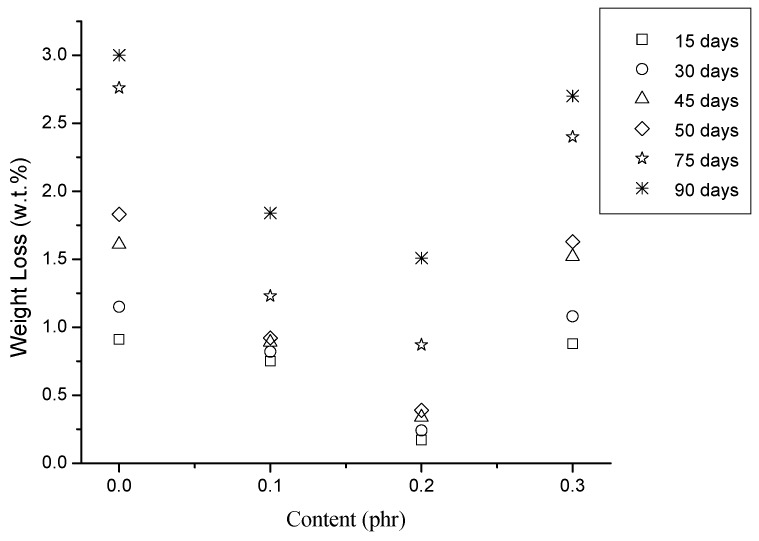
Weight loss of the PLA and PLA/MWCNT-Ag nanocomposites.

The weight loss of the PLA and PLA/MWCNT-Ag nanocomposites at varying hydrolysis times is depicted in Figure 9. Notably, the weight loss of the PLA decreased as the MWCNT-Ag content increased from 0 to 0.2 phr after 30, 60, and 90 days of hydrolysis. This finding might result from water being unable to easily attack the PLA molecules after they are structurally reinforced with MWCNT-Ag. Subsequently, the weight loss of the PLA significantly increased when the MWCNT-Ag content was 0.3 phr; this may be because of the partial MWCNT-Ag aggregation or MWCNT-Ag release, which resulted in defects in the PLA/MWCNT-Ag nanocomposites.

### 3.8. Morphology of PLA/MWCNT-Ag Nanocomposites after Hydrolysis

**Figure 10:**
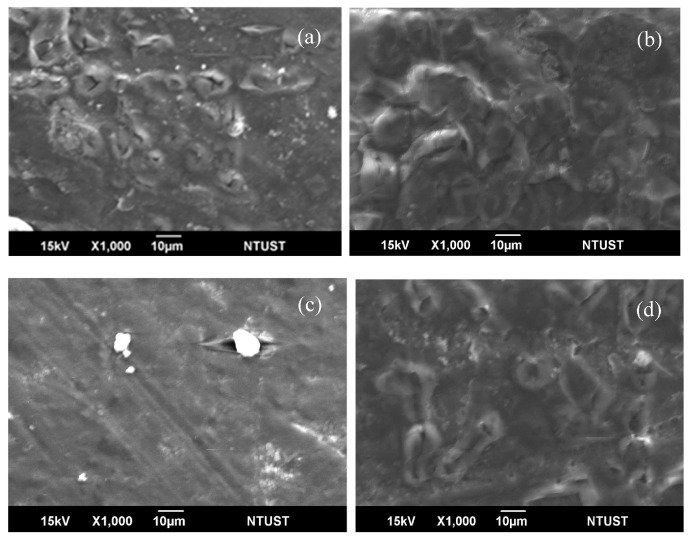
Weight loss of (**a**) PLA; (**b**) PLA/MWCNT-Ag^0.1^; (**c**) PLA/MWCNT-Ag^0.2^; and (**d**) PLA/MWCNT-Ag^0.3^ nanocomposites.

Figure 10 illustrates the surface SEM micrographs of the PLA and PLA/MWCNT-Ag nanocomposite specimens hydrolyzed for 90 days. As expected, a demarcated porous morphology with several connected hydrolysis cavities was observed on the surface of the PLA specimens. However, the size of the hydrolysis-induced voids on the surface of the PLA specimens decreased as the MWCNT-Ag content increased from 0.1 to 0.2 phr. In particular, Figure 10c shows the smooth surface morphology of a PLA containing 0.2 phr of MWCNT-Ag, which may be attributed to the increased packing density and reduced hydrophilic characteristics of the PLA molecules [8,9]. The easy biodegradability of PLA is a disadvantage for its use in bone nails or bone tissue engineering, but the decrease in its biodegradation after loading MWCNT-Ag onto PLA can improve its suitability in biomedical applications [26,27]. However, the number of hydrolysis voids on the surface of the PLA/MWCNT-Ag^0.3^ nanocomposite was much more than that on the surface of the PLA/MWCNT-Ag^0.2^ nanocomposite (Figure 10d), which may have resulted from partial MWCNT-Ag aggregation or MWCNT-Ag release. Furthermore, the packing density [28] of the PLA/MWCNT-Ag nanocomposites decreased because of the high MWCNT-Ag content. 

## 4. Conclusions

An MWCNT-Ag was successfully synthesized using a novel method. The findings presented herein describe the preparation of the PLA- and MWCNT-Ag-based antimicrobial and biocompatible nanocomposites using the melt blending procedure. Overall, this study suggests that MWCNT-Ag loading improves the tensile strength, thermostability, and antimicrobial activity of PLA. The MTT assay demonstrated that PLA/MWCNT-Ag nanocomposites with a high MWCNT-Ag content (≥0.3 phr) are cytotoxic, and the analysis results revealed that the optimal MWCNT-Ag content in the nanocomposite is 0.2 phr. In future research, the nanoAg to MWCNT ratio should be increased to ensure that excellent antibacterial properties are achieved.

## Figures and Tables

**Table 1:** Designations and compositions of the poly(lactic acid) (PLA)/MWCNT-Ag nanocomposites.

Samples	PLA (g)	MWCNT-Ag (g)	BC content (Phr)
PLA	50	0	0
PLA/MWCNT-Ag^0.1^	50	0.05	0.1
PLA/MWCNT-Ag^0.2^	50	0.1	0.2
PLA/MWCNT-Ag^0.3^	50	0.15	0.3

**Table 2:** DSC values of PLA and PLA/MWCNT-Ag nanocomposites.

Samples	*T*_g_ (°C)	*T*_m_ (°C)	*X*_c_ (%)
PLA	67.1	169.3	1.57
PLA/MWCNT-Ag^0.1^	67.1	171.5	15.35
PLA/MWCNT-Ag^0.2^	67.2	171.3	14.98
PLA/MWCNT-Ag^0.3^	67.3	171.5	14.44

**Table 3:** Elemental composition of MWCMT and MWCNT-Ag.

Samples	Element	Weight (%)	Atomic (%)
MWCNT	C	100.00	100
	Ag	-	-
	Totals	100.00	100.00
MWCNT-Ag	C	89.66	98.73
	Ag	10.34	1.27
	Totals	100.00	100.00

## Supplementary Materials

The following are available online at www.mdpi.com/2073-4360/9/3/100/s1, Figure S1: The appearance of (a) MWCNT and (b) MWCNT-Ag, Figure Figure S2: The color of PLA nd PLA/MWCNT-Ag nanocomposites. Figure S3: DTG curves of PLA and PLA/MWCNT-Ag nanocomposites. Figure S4: DSC curves of PLA and PLA/MWCNT-Ag nanocomposites. Figure S5: L929 fibroblasts cultured for control. Figure S6: L929 fibroblasts cultured for 2 d with (a) positive control; (b) negative control; (c) PLA; (d) PLA/MWCNT-Ag^0.1^; (e) PLA/MWCNT-Ag^0.2^; and (f) PLA/MWCNT-Ag^0.3^.
