# Supplementary material for: Antibacterial Property and Cytotoxicity of a Poly(lactic acid)/Nanosilver-Doped Multiwall Carbon Nanotube Nanocomposite
Source: Polymers (Basel). 2017 Mar 10;9(3):100. doi: 10.3390/polym9030100 (PMC6431862; doi:10.3390/polym9030100)
Supplement: Supplementary file 1 [file polymers-09-00100-s001.pdf]

Supplementary materials

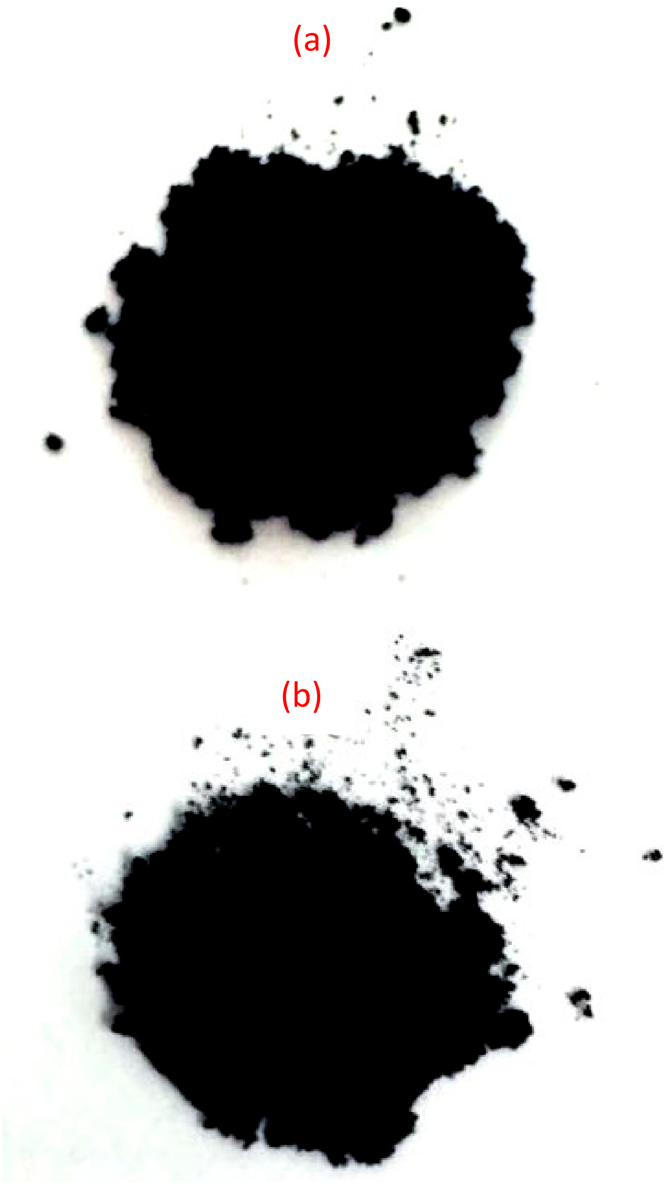

Figure S1. The appearance of (a) MWCNT and (b) MWCNT-Ag

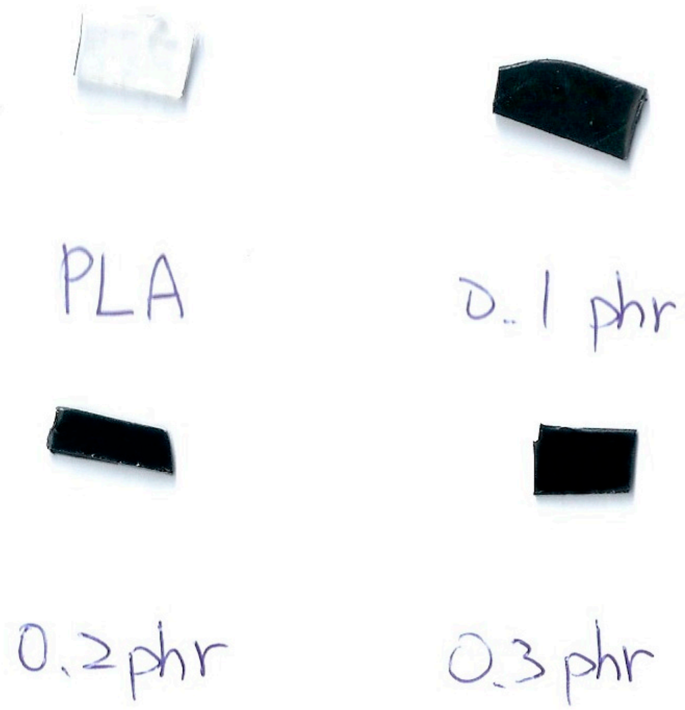

Figure S2. The color of PLA and PLA/MWCNT-Ag nanocomposites.

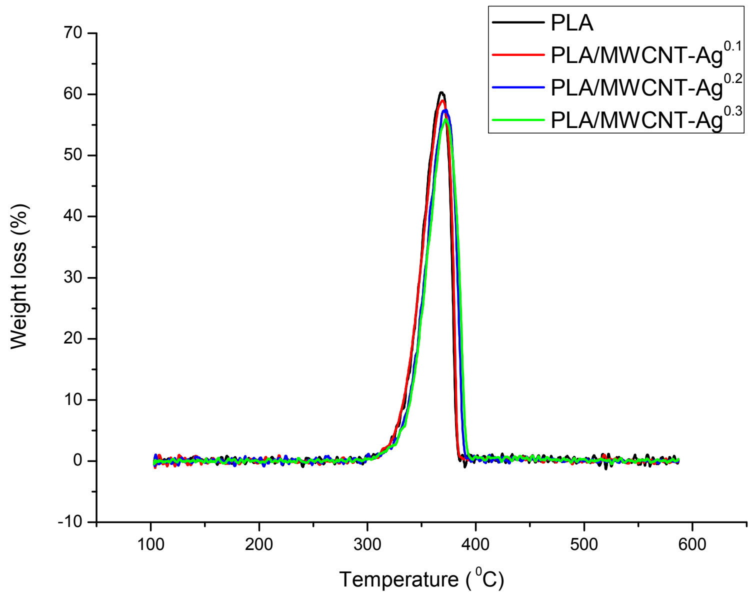

Figure S3. DTG curves of PLA and PLA/MWCNT-Ag nanocomposites.

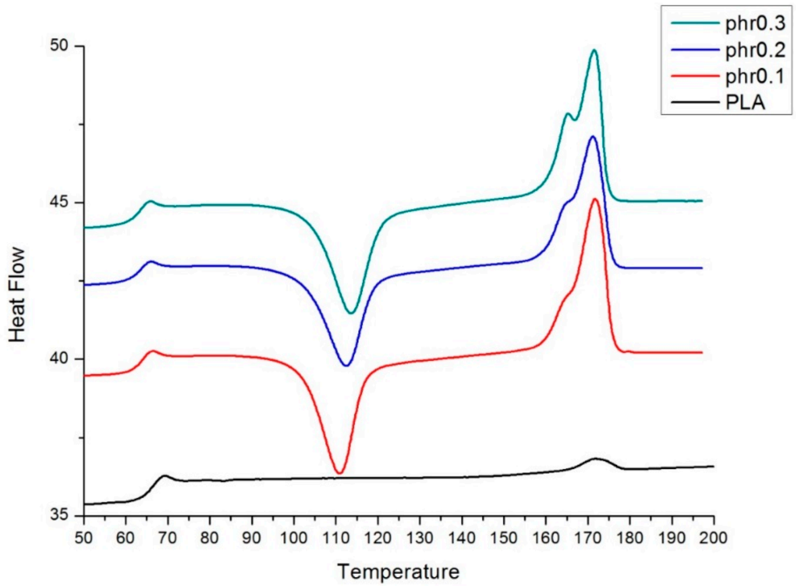

Figure S4. DSC curves of PLA and PLA/MWCNT-Ag nanocomposites.

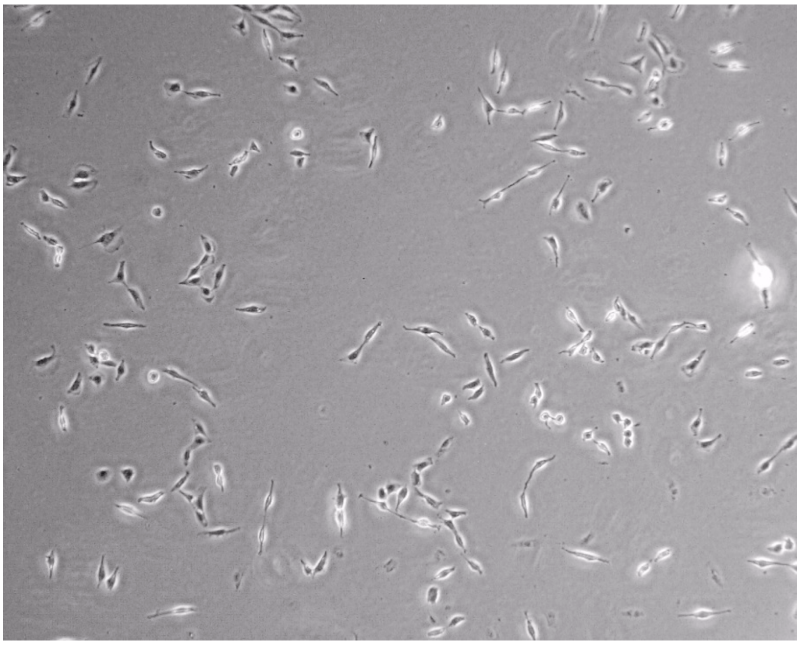

Figure S5. L929 fibroblasts cultured for control

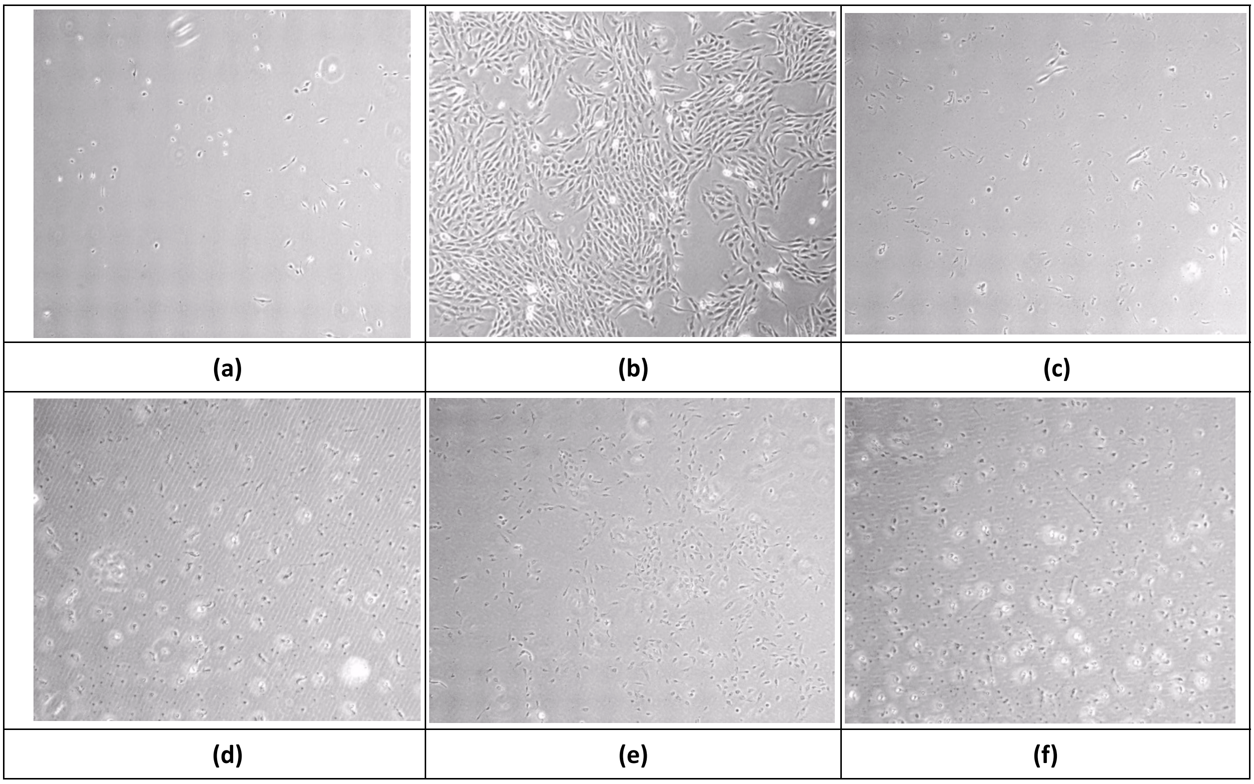

Figure S6. L929 fibroblasts cultured for two days with (a) positive control; (b) negative control; (c) PLA; (d) PLA/MWCNT-$Ag^{0.1}$; (e) PLA/MWCNT-$Ag^{0.2}$; and (f) PLA/MWCNT-$Ag^{0.3}$.
